# Food Webs and Feedbacks: The Untold Ecological Relevance of Antimicrobial Resistance as Seen in Harmful Algal Blooms

**DOI:** 10.3390/microorganisms12112121

**Published:** 2024-10-23

**Authors:** Aabir Banerji, Nichole E. Brinkman, Benjamin Davis, Alison Franklin, Michael Jahne, Scott P. Keely

**Affiliations:** 1 US Environmental Protection Agency, Office of Research and Development, Duluth, MN 55804, USA; 2 US Environmental Protection Agency, Office of Research and Development, Cincinnati, OH 45268, USA; brinkman.nichole@epa.gov (N.E.B.); davis.benjamin@epa.gov (B.D.); jahne.michael@epa.gov (M.J.)

**Keywords:** antibiotic, de novo resistance, horizontal gene transfer (HGT), microbial ecology, trait-mediated indirect interaction (TMII)

## Abstract

Antimicrobial resistance (AMR) has long been framed as an epidemiological and public health concern. Its impacts on the environment are unclear. Yet, the basis for AMR is altered cell physiology. Just as this affects how microbes interact with antimicrobials, it can also affect how they interact with their own species, other species, and their non-living environment. Moreover, if the microbes are globally notorious for causing landscape-level environmental issues, then these effects could alter biodiversity and ecosystem function on a grand scale. To investigate these possibilities, we compiled peer-reviewed literature from the past 20 years regarding AMR in toxic freshwater cyanobacterial harmful algal blooms (HABs). We examined it for evidence of AMR affecting HAB frequency, severity, or persistence. Although no study within our scope was explicitly designed to address the question, multiple studies reported AMR-associated changes in HAB-forming cyanobacteria (and co-occurring microbes) that pertained directly to HAB timing, toxicity, and phase, as well as to the dynamics of HAB-afflicted aquatic food webs. These findings highlight the potential for AMR to have far-reaching environmental impacts (including the loss of biodiversity and ecosystem function) and bring into focus the importance of confronting complex interrelated issues such as AMR and HABs in concert, with interdisciplinary tools and perspectives.

## 1. Introduction

Antimicrobials are substances that are lethal or inhibitory to microbes [1,2]. Alexander Fleming famously discovered what was to be the world’s first mass-produced antimicrobial, the antibiotic penicillin, upon observing *Penicillium notatum* (a species of mold) using it in “chemical warfare” against the bacterium *Staphylococcus aureus* [3,4]. Since then, many other antimicrobials have been discovered in the contexts of similar microbial conflicts, including antibiotics/antibacterials from amoebae [5], amebicides and antifungals from bacteria [5,6], and antiprotozoals and anthelmintics from red algae [7,8]. Unfortunately, worldwide, antimicrobials such as these have increasingly been giving rise (evolutionarily) to microbes that can survive and reproduce in their presence [9]. This microbial ability is known as “antimicrobial resistance” (AMR).

AMR has, for decades, been the focus of international multidisciplinary research efforts, not only in clinical settings [10,11] but also in residences [12,13], factories [14], farms [15,16], research laboratories [17], and the environment [18,19]. Historically, it has been framed as an issue of epidemiology and public health. Its potential impacts on the environment (i.e., on ecosystem health and services) have rarely been considered, let alone characterized or quantified. While there have been recent efforts to foster “antimicrobial stewardship” [20,21,22] and leverage the efficacy of “One Health” methodologies, even these have mostly been focused on protecting humans and other organisms of economic interest from multi-drug-resistant pathogens [23,24,25,26,27,28,29] and on controlling anthropogenic drivers of AMR, such as wastewater inputs [25,29,30,31,32]. Reviews of AMR in the environment have acknowledged the environment as an arena for both the circulation of already-resistant bacteria and the evolution of de novo resistance (novel forms of AMR or novel AMR–microbe combinations) [33,34,35] but still tend to stress the importance of distinguishing “clinically relevant” microbe–AMR combinations from the multitude of other varieties detectable in the environment [36,37].

Some experts have posited that it is unrealistic to expect AMR to pose environmental risks, let alone risks dire enough to justify new research and mitigation expenditures [30,35]. They argue that AMR is a defense against antimicrobials and therefore would not even reveal itself (phenotypically) in the absence of antimicrobials or related contaminants [38]. By this logic, microbes’ possession of AMR cannot lead to the death or inhibition of other organisms (loss of biodiversity) and, thus, should not be treated as an environmental issue in itself but, rather, as a symptom of the problem of antimicrobial pollution or an intensifier of the problem of infectious disease. AMR, if anything, might even safeguard against the loss of biodiversity in natural ecosystems [35], because, without it, anthropogenic inputs of antimicrobials would kill or inhibit “beneficial” microbes responsible for processes such as biogeochemical cycles [39,40], lower food web activity [41], the biodegradation of pollution [42], and the metabolism and immune responses of plants and animals [43,44]. A few researchers have also argued that the rise and spread of AMR beyond the point of input of antimicrobial pollution is likely to be limited, since the environment naturally self-remediates contaminants through physical dilution [45,46], biodegradation [47], and retention within clay and dead biomass (“necrobiome detoxification”) [48].

**Figure 1:**
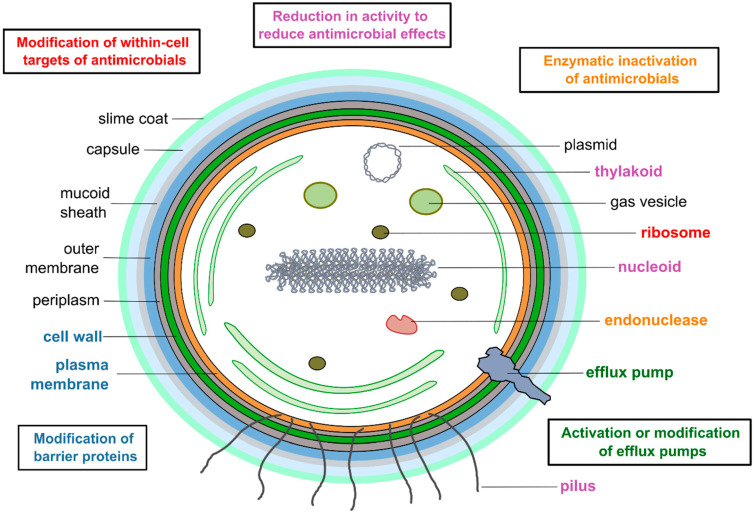
Cyanobacteria can exhibit all the known mechanisms of AMR found in other bacteria. These relate to cell physiology, regardless of whether they are general stress responses [81] or specific defenses against specific antimicrobials [82]. Methylation of the ribosomes, for example, can create AMR against ribosome-targeting antimicrobials.

As these fellow scientists have implied, it is important to recognize (1) that ecosystems can be resilient and (2) that ecologists and microbiologists should not conflate the problem of AMR with that of antimicrobial pollution—or consider AMR “bad” for humans and the environment across all scenarios. However, it may be premature to dismiss the possibility of AMR having ecological relevance. Traits analogous in function to AMR have long garnered attention as potential threats to biodiversity. These traits have also been evaluated as tools for environmental monitoring, environmental mitigation, climate change preparation, and/or the enhancement of agricultural yield. They include various forms of stress resistance within genetically modified organisms [49,50,51], invasive plants [52,53,54], invasive pathogens [55,56,57,58,59], agricultural weeds [60,61], pestilent insects [62,63], and hybrids born of species introgression [64,65]. AMR, through its underlying variations in microbial physiology [66] (e.g., modifications of barrier proteins, efflux pumps, enzymatic activity, and within-cell targets of antibiotics; Figure 1), can theoretically alter how microbes interact with members of their own populations, with co-occurring species, and with their non-living environment, just as it influences how the microbes interact with antimicrobials. The environmental consequences of these effects may be significant, depending on what ecological roles the microbes play, whether they have many or few ecological relationships, and whether they interact strongly or weakly in those relationships. Microbes that have caused landscape-level environmental issues worldwide would be especially worth investigating for such AMR side effects. If AMR exacerbates the microbes’ harmfulness, then this would be even more reason to keep its occurrence in the environment in check. If it reduces their harmfulness, then its prevalence or expression could potentially be manipulated to help control these microbes.

A notorious example of such microbes are the cyanobacteria that form freshwater harmful algal blooms (HABs). HABs are dense assemblages that are often large enough to be detectable via satellites [67]. They present numerous human and environmental health risks in freshwater aquatic ecosystems—including high concentrations of toxins and skin irritants [68,69,70], areas of low oxygen availability [71], dramatic shifts in pH [72], biofouling [73], catastrophic regime shifts in phytoplankton and zooplankton [74,75], and outbreaks of waterborne diseases associated with microbial symbioses and planktonic decay [76,77]. AMR’s effects on HABs might—for better or worse—alter human access to ecosystem services such as safe drinking water, irrigation, fishing, and recreation. They might also extend beyond the aquatic realm to terrestrial habitats such as forests and urban areas, via shifts in the feeding behaviors of seabirds [78], raptors [79], and aquatic mammals [80].

## 2. Scope and Methodology of This Review

To uncover evidence of AMR affecting HABs (i.e., hastening or slowing their onset or increasing or decreasing their frequency, severity, or duration), we used the online search engine Google Scholar to perform a literature review of peer-reviewed scientific journal articles published in English during the timeframe of 2004–2024. Using the search terms “antimicrobial”, “cyanobacterium”, and “bloom” (allowing for plural forms, acronyms, and inexact matches), we compiled over 13,000 unique results. We then screened these results (initially, based on their titles and abstracts) to exclude studies that did not pertain to cyanobacterial HABs in inland freshwater ecosystems (e.g., lakes and rivers) and to AMR conferred by known “antimicrobial resistance genes” (ARGs) [38]. Although other aquatic habitats (including marine, inquiline, and artificial) and other means of acquiring AMR (e.g., via cooperation with other species) are equally relevant to the topic [83,84,85], this screening was necessary to ensure tractability. Of the approximately 400 remaining studies, none were explicitly designed to determine whether AMR affects HABs and/or the dynamics of HAB-afflicted aquatic food webs. Nevertheless, among them, we found multiple studies reporting AMR-associated changes in HAB-forming cyanobacteria and co-occurring microbes that pertained directly to HAB timing, toxicity, and phase. In the sections that follow, we synthesize what these studies reveal.

## 3. Current Understandings of the Relationship between AMR and HABs

### 3.1. Overlapping Features and Contexts

**Figure 2:**
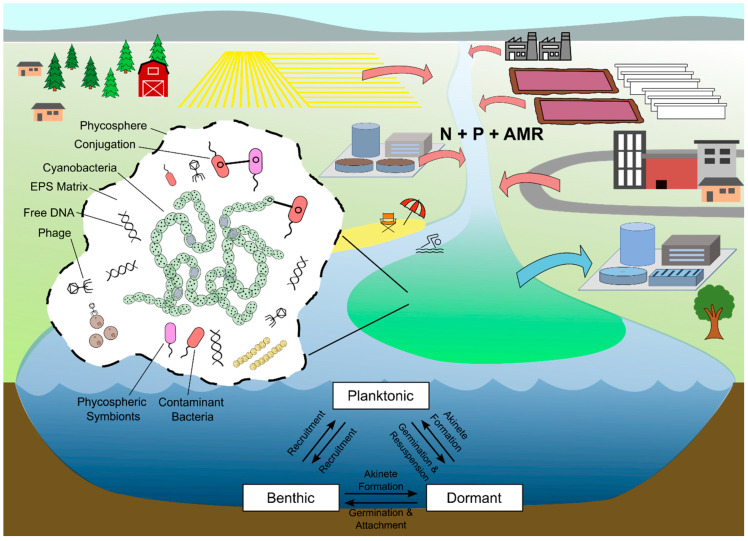
Watershed-level depiction of how AMR and HABs overlap and interrelate in terms of where they occur, what anthropogenic inputs promote them, the kinds of feedbacks they are involved in, and the sorts of exposure risks they can create.

Despite being viewed as separate concerns, AMR and HABs share many similarities and connections (Figure 2). Both occur where exposure risks are imperative to address (e.g., drinking water sources and public beaches) [86,87] and where microbes such as human pathogens are likely to co-mingle and accumulate (e.g., catchments of wastewater and agricultural runoff) [88,89,90,91]. Both are facilitated by global climate change [92,93] and “cultural eutrophication”—the process by which nutrients from anthropogenic sources interfere with ecological community dynamics and biogeochemical cycles [94,95,96]. Both have called for the identification and enumeration of microbial species, the tracking of microbial activity, and the profiling of microbial traits to protect the environment and human health [97,98]. Both can also be linked at the cellular level [99,100], as HAB-forming cyanobacteria often possess ARGs and can potentially exchange ARGs with other microbes (e.g., via plasmids) [101,102]. These same cyanobacteria host a diversity of heterotrophic microbes within the mucus that encapsulates their cells (their phycospheric symbionts) [103,104], which often likewise possess ARGs and the ability to exchange ARGs with other microbes [99,105]. Whether or not ARGs in this context can exacerbate HABs, they can exacerbate water quality issues tied to HABs, such as the cyanobacterial contamination of crops [106,107] and hospital dialysate [108] and the cyanobacterial colonization of human respiratory tracts [109]. Reciprocally, the occurrence of HABs may increase ARG diversity within planktonic microbial communities [110].

Accordingly, some of the same or similar methods of control are being used to monitor, prevent, and mitigate AMR and HABs [111,112,113] (Table 1). Peroxide, for example, which is commonly used as an algaecide, has been shown to also be effective at killing or inhibiting multi-drug-resistant bacterial pathogens [114,115,116]. Certain antibiotics have likewise been found to be effective as algaecides against cyanobacteria [117]. If the severity of HABs and the prevalence of AMR are positively correlated, then methods of controlling HABs may double as methods for controlling AMR, which would resolve two water quality issues for the price of one. On the other hand, should the increasing severity of HABs come with a decreasing AMR prevalence (or vice versa), then aquatic resource managers may find themselves in the unenviable position of having to optimize the balance of contrasting risks [118,119].

### 3.2. HABs as Biofilms and Hot Spots for AMR Evolution

Though rarely referred to as such, HABs are essentially large, floating biofilms: communities of aggregated microbial cells embedded in a self-produced matrix of macromolecules (“extracellular polymeric substances” [154]). These often complex, three-dimensional structures provide their constituents with evolutionary advantages—e.g., joint defenses against ultraviolet radiation, extreme temperature, extreme pH, high salinity, low nutrients, and, indeed, antimicrobials [155,156,157]. HAB-constituent species that do not already possess ARGs can acquire them through genetic mutation and horizontal gene transfer (HGT) [158,159,160,161]. Within HABs, just as they are within other kinds of biofilms, the likelihoods of mutation and HGT is higher compared to that among dispersed (non-aggregate) populations of microbes. This is due to the accrual and arrangement of cells and the HABs’ alteration of the ambient pH and oxygen concentration, which increase the frequency of co-occurrence and contact-dependent interactions among compatible microbial species [162,163,164]. HABs may also increase the rates of the appearance and exchange of ARGs by promoting plasmid stability via the induction of so-called “mafia traits” that are encoded on mobile genetic elements [165,166] and via the release of outer membrane vesicles (OMVs) [158], both of which are triggered by quorum sensing and other forms of cell-to-cell signaling [167,168].

Furthermore, some of the toxins and non-toxic secondary metabolites produced in HABs (e.g., alkaloids, polyketides, terpenes, and polyphenols) have been reported to have antimicrobial properties. While these have garnered attention as potential alternatives to established pharmaceuticals [169,170], there is evidence to suggest that they, too, may select for ARGs [171,172,173]. This could make AMR more common or more versatile in toxic HAB-forming cyanobacteria and their phycospheric symbionts than in co-occurring microbial competitors, which would explain why antimicrobial pollution has been shown to increase the likelihood of HABs [100,174]. The AMR-exhibiting HAB constituents would survive and reproduce, while their AMR-lacking competitors would be killed or inhibited by the antimicrobials, allowing the former to gain exclusive access to previously contested resources, as well as to the resources that arise from their competitors’ lysed remains [100,174,175,176,177]. There is also evidence that cyanobacterial toxins such as microcystin-LR promote the HGT of ARGs by regulating gene systems involved in microbial conjugation, stimulating the formation of reactive oxygen species (ROS), and increasing cell membrane permeability [178].

## 4. How AMR Affects HAB Dynamics and Severity

### 4.1. Effects on Timing

Factors that suppress the growth of HAB-forming cyanobacteria can cause HABs to form more gradually, later in the season, or with lower cell density and spatial coverage [179,180]. They can also lead to HABs simply not forming at all, due to HAB-constituent cells being crowded out by more proliferative competitors [181] or being more thoroughly grazed by consumers they could have otherwise deterred or over-sated [182,183]. ARGs being associated with such tradeoffs is well documented in pathogens and some model bacterial populations [184,185,186,187,188,189,190,191]. Some ARGs, for instance, work by modifying cellular transport mechanisms, which reduces the cell’s efficiency at sequestering nutrients and increases its vulnerability to phage attachment [192]. Merely possessing ARGs may increase the cell’s demand for nutrients, energy, and intracellular space, as these resources are required for accumulating and replicating DNA [193,194,195,196,197]. On the other hand, having slower growth and a lower population density can also improve the chances of persisting in the face of nutrient limitation [198,199,200] and evading consumers [201,202]—especially if the consumers rely on density-dependent cues [203,204].

Several studies within our scope suggest that cyanobacteria benefit ecologically from ARGs and pay biological fitness costs to do so. San Millan et al. (2014) [162] found 48 different cyanobacterial genomes in GenBank (representing multiple genera) that tend to house coexisting ARG-containing plasmids (with no indication of plasmid size affecting plasmid presence)—but also found that these genomes tended not to house more than two such plasmids at a time. Cassier-Chauvat & Chauvat (2015) [205] highlight that, in at least a few ARG-possessing cyanobacterial genera (including *Synechocystis*), AMR doubles as an adaptive response to oxidative and heavy metal stresses. Vogel et al. (2017) [206] reported lower intrinsic growth rates in *Synechococcus* sp. PCC 7002 compared to wildtype *Synechococcus* upon the artificial insertion of kanamycin resistance genes—a common form of ARGs. Whether these apparent tradeoffs influence the dynamics of HABs still has to be examined empirically.

### 4.2. Effects on Toxicity

Identifying and distinguishing ARG effects on cyanobacterial toxicity is a complicated challenge. Aquatic environments house multiple factors that can affect both the synthesis of toxins and the susceptibility of organisms to toxic effects. Further complicating the matter is the present lack of knowledge regarding cyanobacterial toxins and the subjectivity with which researchers classify “toxins” and “antimicrobials”. Even for microcystins (the best-studied class of cyanobacterial toxins), at least 246 known variants have been isolated, of which only a few have been characterized toxicologically [207,208]. Certain toxins are used by cyanobacteria to kill or inhibit other microbes [209] and by other microbes to kill or inhibit cyanobacteria [210,211,212], which would rightly inspire some researchers to regard these toxins as antimicrobials (and, in turn, regard microbial tolerance of the toxins as AMR).

Nonetheless, in the genome of *Microcystis aeruginosa*, separate ARGs and toxin synthesis genes have been distinguished from one another and found to co-occur. This allows for a straightforward evaluation of their relationship and of the potential tradeoffs in their expression. Wu et al. (2022) [213] found that ARGs (specifically, sul1, sul2, tetW, and tetM) were positively correlated with a microcystin synthetase gene (mcyA-J). While this genetic linkage is insufficient to infer what happens at the level of expression (i.e., how, if at all, the genes’ respective gene products intermingle), its being positive suggests that inherent tradeoffs between AMR and toxin production must be minor or somehow counter-balanced in *M. aeruginosa*. Since other HAB-forming cyanobacteria (e.g., *Planktothrix agardhii*) [214] possess pathways homologous to those of *M. aeruginosa*, tradeoffs between AMR and toxin production are perhaps minor or counter-balanced in them, as well.

### 4.3. Effects on Phase

Many HAB-forming cyanobacteria have multi-phasic life cycles that encompass transitions between dormancy and activity, benthic and planktonic distributions, and dispersed and aggregate populations or growth forms (Figure 2). At the onset of HABs, these cyanobacteria, having emerged from dormancy within benthic sediment or colonized their aquatic habitat from elsewhere, become abundant and metabolically active. They generate and respond to quorum-sensing cues; form colonies, biofilms, and microbial consortia; and engage in mutualistic and antagonistic exchanges [215,216,217]. At the end of HABs, when the HABs dissipate, the cyanobacteria senesce or enter dormancy, due to factors such as starvation, disease, and changes in season. Initiating and sustaining these different phases requires various criteria to be met. For example, overwintering in a dormant state as akinetes [218] or in a fortified benthic or planktonic form [147] requires cold tolerance and adequate reserves of nutrients and energy. Forming colonies, filaments, and benthic mats requires not only growth and proliferation but also exchanges of chemical signals for cell-to-cell coordination and compartmentalization [219,220,221].

ARGs may influence these HAB-constituent attributes in various ways. For instance, they can alter cell membrane features associated with akinete viability [222,223] and the release of volatile organic compounds (VOCs) [224,225]. VOCs such as geosmin and β-cyclocitral are used by cyanobacteria not only to initiate biofilm formation and communal AMR mechanisms with other species [84,150] but also to interfere with competing phytoplankton, to repel or signal poor nutritional value to grazers [221,226], and to prime toxin synthesis [224,225]. These same VOCs can also add to the severity of HABs by causing taste and odor issues and disrupting various physiological functions in various organisms [221,227]. Although no study within our scope reported ARG effects on overwintering and aggregation in HABs, some did report evidence of ARGs affecting prerequisite or complementary cyanobacterial adaptations. Yang et al. (2008) [228] found that an occasionally HAB-forming strain of *Synechocystis* [229] gains its tolerance to daytime cold temperature (“chill-light tolerance”) from its natural synthesis of the antimicrobial alpha tocopherol and putative possession of the corresponding ARGs [230]. Others have shown that ARGs can affect the transmission and receipt of VOCs in heterotrophic bacteria, including some that might be found within the phycospheres of HAB-forming cyanobacteria [219,220,221,231,232,233].

### 4.4. Effects on Indirect Interactions

An “indirect” interaction is where one species affects another species by changing the population density, morphology, physiology, or behavior of a third species [234,235]. There is a long history of the applied use of indirect interactions in aquatic remediation and restoration (e.g., stocking fish to control phytoplankton via fish consumption of zooplankton) [236,237]. Because indirect interactions stem from direct (pairwise) interactions, it is a given that species’ traits influencing the latter must also influence the former. That influence, as previously alluded to, can even extend beyond the aquatic realm to terrestrial species such as bald eagles (*Haliaeetus leucocephalus*) [238], as well as transform important landscape features of the habitat (e.g., organic matter and dissolved oxygen at the surface of benthic sediments and the optical clarity of the water column) [239]. Natural resource managers and public health officials are also cognizant of indirect interactions due to the possibility of the interactions either enhancing control efforts or creating unintended consequences. Even if due to factors such as pollution, habitat alteration, and climate change, rather than to targeted removal efforts, increases in the prevalence of AMR and losses of specific groups of microbes may exacerbate existing microbial threats and create new ones.

In the context of HABs, there are numerous indirect interactions to consider. Examples include HAB-forming cyanobacteria benefiting co-occurring phytoplankton by their deterrence of grazers or harming other phytoplankton by inciting grazers to feed preferentially on phytoplankton with greater nutritional value [221,226]. There are also intriguing examples that involve the relationship between HAB-forming cyanobacteria and aquatic fungi. Multiple studies have established that fungal parasites of HAB-forming cyanobacteria can increase the ability of zooplankton to feed on and assimilate their cyanobacterial hosts [240,241,242]. Several fungicides associated with agricultural runoff (namely, tebuconazole, azoxystrobin, and itraconazole) have been found to promote HABs by killing or inhibiting such parasites [243]. Conversely, it can be fungi that interfere with the zooplankton consumption of HAB-forming cyanobacteria and the phytoplankton that facilitate it. Sánchez et al. (2019) [244] found that consuming a mix of toxic HAB-forming cyanobacteria and green algae prevents the cladoceran *Daphnia dentifera* from being infected by fungal parasites (genus *Metschnikowia*) and increases offspring production in already-infected hosts. This creates a level of predation pressure on both phytoplankton prey that is higher than what either would have experienced in the absence of the other (a phenomenon which ecologists refer to as “apparent competition”) [245].

Because of such ecological relationships, siloed efforts to control aquatic fungi may inadvertently promote HAB-forming cyanobacteria, and efforts to control HABs may inadvertently promote aquatic fungi. This would be analogous to a medical complication in clinical settings, wherein the use of antimicrobials (e.g., vancomycin) to treat bacterial infections can inadvertently promote invasive fungal infection and systemic bacterial co-infection [246]. No studies within the scope of our review have uncovered how AMR-related changes in physiology, be they in the cyanobacteria or the fungi, might affect the relationships between these organisms and the outcome of control efforts.

## 5. Future Directions

HABs and AMR are each pressing concerns in their own right but are also interrelated at many levels (the levels of genes, cells, populations, communities, and ecosystems, as described in the previous sections). This interrelatedness must be accounted for along with environmental impacts if one is to fully assess even their respective economic tolls. Thus, thoroughly addressing either concern ultimately requires the well-coordinated handling of both [110], with the application of systems thinking [29,247,248]. Advancements in environmental monitoring, prevention (pre-crisis), and mitigation (post-crisis) are all necessary and fair game in fulfilling this objective.

### 5.1. Environmental Monitoring

New technologies are increasingly making it cost-effective to comprehensively sample and survey ecosystems of concern over space and time, as well as to process immense quantities of multivariate data. Among these technologies are remote sensing tools, high-throughput genomic and bioinformatic pipelines, and artificial intelligence-based analyses and predictions, which have already been leveraged or proposed for the purposes of monitoring HABs and AMR (Table 1). Environmental DNA (eDNA) analysis is particularly useful. However, some advanced technologies are still cost-prohibitive and/or must overcome other hurdles besides costs before they can be utilized more widely and routinely. The latter hurdles may include bureaucratic limitations requiring the cooperation of multiple groups at multiple levels. They may also include the conceptual challenge of defining and obtaining reliable baselines of comparison (e.g., when evaluating risks, damages, and the success of control efforts).

For HABs, differences arise among locations and over time in (1) HAB occurrence, (2) public awareness/perception of HABs, (3) the comprehensiveness and precision of HABs monitoring, and (4) the (socioeconomic) community capacity for HABs monitoring. Each of these may make comparisons across space or with the past misleading. Similarly, for AMR, habitats deemed to have little to no pollution may nevertheless be rich in natural sources of ARGs and antimicrobials due to other conditions. Natural resource management in light of these concerns would almost certainly benefit from continued and expanded investment in social science investigations, public outreach, public engagement, and collaborative partnerships among government and non-government institutions [249,250] to assess status and effect change. Additionally, a particularly novel avenue of progress that has recently gained some traction among environmental microbiologists is the treatment of either DNA and RNA in general or particular kinds of genetic machinery (e.g., integrons) as environmental pollutants [251]. The adoption of such targets as environmental monitoring indicators might enable scientists to develop new standardized criteria and thresholds for safeguarding aquatic ecosystem services (e.g., drinking water, recreational water, wastewater, and sustainable fisheries and aquaculture). These could, in turn, accommodate or be modified to accommodate toxin synthesis genes, nutrient metabolism genes, and ARGs in HAB-forming cyanobacteria and other microbes [19].

### 5.2. Prevention

Current approaches to preventing the rise and spread of HABs and AMR in the environment mostly revolve around curtailing and removing anthropogenic inputs of substances favoring nuisance microbial characteristics (e.g., growth-limiting nutrients; Table 1). These are and will continue to be important but have proven insufficient to stop HABs and AMR-related problems from occurring. More targeted and deployable methods have recently been proposed for the removal of ARGs and nutrients from eutrophic aquatic ecosystems—including some that incorporate the use of algal-bacterial consortia [252]. Also under consideration are “integrated” methods (i.e., ones that combine physical, chemical, and/or biological control) [253], such as the manipulation of microbial communities to oust HAB-forming cyanobacteria and AMR-exhibiting pathogens or prevent them from establishing. Takeuchi et al. (2021) [254], for example, found that the use of certain combinations of nutrients and substrate (in the form of culture media) promoted the growth of bacteria antagonistic to *Flavobacterium psychrophilum*, the cause of Rainbow Trout Fry Syndrome (RTFS) and Bacterial Coldwater Disease (BCWD) in freshwater fish.

The manipulation of microbial communities to prevent HABs and AMR could also be achieved through means such as the disruption of quorum sensing and cell-to-cell adhesion [255,256] or the reconstruction of an overwintering habitat, inclusive of planting and/or re-planting non-nuisance native benthic microbes and plants [153,257]. Similar integrated approaches have been used widely and effectively to address issues such as the spread of invasive species and new and re-emerging infectious diseases [151].

### 5.3. Mitigation

A highly anticipated advancement that might enable natural resource managers to combat HABs and AMR in concert even as they occur is the use of phages for the biological control of HAB-constituent microbes and pathogens [258,259]. Phages (and viruses in general) are considered less likely than many other candidate biological control agents to generate non-target effects or spread beyond the area of application, due to their potential specificity and general inability to reproduce outside of living hosts [260,261]. However, they often have higher mutation rates than even their microbial hosts, which can give rise to pestilent phages or ones that coevolve with their targets in such a way as to become ineffective as biological control agents [262]. Depending on how they are deployed, they may disrupt the microbiomes of non-target eukaryotes [263]. Moreover, some have been found to contribute to AMR in the environment [264] and to alter the competitive interactions of HAB-forming cyanobacteria in unexpected ways [265]. Successfully unlocking their potential as biological control agents, given these concerns, may require sophisticated tactics such as using genetic engineering to tailor their effects on microbes in the environment [266,267] or restricting their sphere of influence to contained environments such as bioreactors within treatment plants [268,269,270].

## 6. Conclusions

Humans rely on antimicrobials for treating and preventing infections among humans [271], pets [272], livestock [273], and crops [274] and for purging microbial contaminations from the live cultures used in industry, culinary processes, and research [275,276,277]. This, for decades, has motivated researchers to investigate AMR as an epidemiological and public health problem, separate from issues impacting the environment. However, in investigating its effects on microbe-driven environmental disturbances, we have found that AMR can have impacts on the environment as well as human health. The freshwater cyanobacterial HABs that we have focused on in this review are conspicuous examples of such disturbances, ones that scale to the landscape level and beyond [67,79,80], but they are not unique. Others include the sorts of HABs formed by red seaweed [278], silicoflagellates [279], diatoms [280], dinoflagellates [281], haptophytes [282], or euglenophytes [283]. Such typically marine and benthic HABs have been found to dramatically impair coastal ecosystems and related human activities, including fisheries, tourism, aquaculture, and restoration. There are also fungal outbreaks of chytridiomycosis, which have caused the extinction of at least 90 different amphibian species and endangered over 400 others [284,285]; proteobacterial outbreaks of epitheliocystis, which are an ongoing cause of global fish declines [286]; and percolozoan outbreaks of Primary Amebic Meningoencephalitis, which have caused mortality in over 95% of human cases [287,288,289]. AMR may be akin to the generalist features ascribed to macroscopic invasives—i.e., a trademark of “weedy” microbes, which should warrant the same level of attention, as it might enable microbes to become invasive [290]. Even if it had been safe to assume that natural ecosystems tend to be harsher environments for microbes that exhibit AMR than for those that do not, this superficially preferable scenario can still give rise to adverse ecological outcomes because of indirect interactions. In summary, the ecology of AMR is still rife with knowledge gaps, as well as opportunities for innovation in natural resource management and the safeguarding of public health. Interdisciplinary research and development are required on many fronts to tap its potential and avoid unintended consequences of control efforts.

## Figures and Tables

**Table 1:** Side-by-side comparison of approaches to tracking, preventing, and mitigating AMR and HABs.

AMR	HABs
•Using cell culture, molecular assays [120], and advanced spectroscopy [66] to track ARGs and AMR determinants across clinical, agricultural, retail, and environmental [111,112,113] settings [121,122].	•Using microscopy, molecular assays, and remote sensing to track HAB-forming cyanobacteria and cyanobacterial toxin synthesis genes in the environment [98,123,124].
•Quantifying socioeconomic impacts of AMR via surveys and public record analysis [125].	•Quantifying socioeconomic impacts of HABs via surveys and public record analysis [126].
•Promoting wastewater and stormwater treatment methods that limit fecal indicator species and antimicrobials in effluent [20,127].	•Promoting wastewater and stormwater treatment methods that limit nutrients and HAB-favoring pesticides in effluent [118,128].
•Supporting best management practices for administering antimicrobials [20,129,130,131].	•Supporting best management practices for applying potentially HAB-fueling nutrient fertilizers [128].
•Maintaining wastewater, stormwater, and sewage treatment infrastructures, as well as expanding green infrastructures, to intercept and contain microbial/antimicrobial pollution [118,119].	•Maintaining wastewater, stormwater, and sewage treatment infrastructures, as well as expanding green infrastructures, to intercept and contain nutrient pollution [118,128].
•Socially implementing best hygiene practices to disrupt disease transmission [132] and improving compliance in hospitals [133].	•Socially implementing cleaning protocols to disrupt the transport of HAB-forming cyanobacteria by boat hulls, ballasts, gear, and boots [134,135].
•Developing vaccines, prophylactics, and alternative control strategies in place of antimicrobials [136,137,138].	•Developing alternative control strategies in place of algaecides [111,139,140].
•Developing means to physically or biochemically degrade antimicrobial pollution [141,142].	•Applying substances like modified clay to flocculate dispersed HAB-forming cyanobacteria [143] and altering reservoir hydrodynamics to physically flush out HABs or disrupt cyanobacterial dominance [144].
•Developing or bioprospecting novel antimicrobials [145].	•Developing or bioprospecting novel algaecides [146] or applying existing algaecides in novel ways [147]. This may include using antibiotics to kill or inhibit cyanobacteria without causing harm to eukaryotic phytoplankton and zooplankton [117].
•Maintaining and upgrading non-pharmaceutical methods such as UV-irradiation, ozonation, and chlorination to disinfect/degrade microbes in drinking water and wastewater [148].	•Maintaining and upgrading methods such as UV-irradiation and granulated/particulate-activated carbon to neutralize/remove cyanobacterial cells and toxins in drinking water and wastewater [128].
•Applying quorum-silencing/quenching agents to disrupt pathogenic virulence [149] and AMR [150].	•Applying quorum-silencing/quenching agents to disrupt HABs [151].
•Administering probiotic supplements following antibiotic treatments in clinical patients to restore gut microbiome biodiversity [152].	•Planting/re-planting submerged vegetation or benign phytoplankton to restore aquatic microbial biodiversity [151,153].

## Glossary

Term	Definition	References
Antibiotic	An antimicrobial that targets bacteria. The term is sometimes used interchangeably with “antibacterial” but is not to be confused with the more general categorizations of “antimicrobial”, “biocide”, and “poison”.	[291,292]
Conjugation (Microbial)	The process by which donor microbes transfer DNA to compatible recipient microbes through sexual exchanges involving tube-like pili.	[159]
Ecosystem	A location housing a community of living organisms that interact with each other and their non-living environment, its spatial boundaries and sphere of influence being defined ad hoc based on case-specific objectives.	[293,294]
Ecosystem health	The potential of an ecosystem to retain its organizational structure, biodiversity, and biogeochemical functions under stress (integrity) and to return to that state following disturbance (resilience).	[295,296,297]
Ecosystem services	Ecosystem features or benefits that create interdependencies between ecosystem health and the socioeconomic needs and wants of humans.	[298,299]
Epidemiology	The study of the biological, physical, chemical, and socioeconomic determinants of disease incidence and distribution among populations and the applications of its findings for disease prevention and control.	[300,301,302,303]
Intrinsic Growth Rate	The highest rate at which individuals of a species can theoretically reproduce (maximum per capita population growth rate or doubling time, i.e., birth rate without death and inhibition).	[304]
Introgression (Species)	The acquisition of genetic variation in a species’ population from another species’ population through mating.	[65]
Microbe	Any organism too small to be seen by the naked human eye. The term refers to numerous bacteria, archaea, protozoa, and algae, as well as certain animals, such as rotifers, cladocerans, tardigrades, and *Demodex* mites. Infrequently, it is also used to refer to organisms that, despite being single-celled or in the same taxonomic clade as well-established microbes, are visible to the naked human eye, such as the green alga *Valonia ventricosa* (a root-fouling mangrove epibiont that can grow up to 5 cm in length) and the bacterium *Epulopiscium fishelsoni* (a cigar-shaped gut symbiont of the brown surgeonfish, *Acanthurus nigrofuscus*, that can grow up to 600 µm in length—approximately seven times the width of a human hair).	[305,306,307,308]
Phage	A virus that targets non-eukaryotic microbes. Phages that target cyanobacteria are commonly referred to as “cyanophages”, whereas those that target other bacteria are called “bacteriophages”. Viruses that target other viruses are called “virophages”.	[309,310,311]
Quorum Sensing	The process of chemically mediated cell-to-cell communication that allows bacteria to regulate their gene expression in response to changes in population density.	[312]
